# Preparation and Properties of Lightweight Amphiphobic Proppant for Hydraulic Fracturing

**DOI:** 10.3390/polym16182575

**Published:** 2024-09-12

**Authors:** Guang Wang, Qinyue Ma, Longqiang Ren, Jirui Hou

**Affiliations:** 1 Unconventional Petroleum Research Institute, China University of Petroleum-Beijing, Beijing 102249, China; wangguang@zgsydxbj.wecom.work; 2 Bei Jing Kun Lun Long Yuan Petroleum Exploitation Technology Co., Ltd., Beijing 102200, China; 3 Liaoning Longyuan Sand Industry Co., Ltd., Fuxin 123200, China

**Keywords:** lightweight, proppant, amphiphobic, conductivity, placement

## Abstract

The wettability of the proppant is crucial in optimizing the flowback of fracturing fluids and improving the recovery of the produced hydrocarbons. Neutral wet proppants have been proven to improve the fluid flow by reducing the interaction between the fluid and the proppant surface. In this study, a lightweight amphiphobic proppant (LWAP) was prepared by coating a lightweight ceramic proppant (LWCP) with phenolic resin, epoxy resin, polytetrafluoroethylene (PTFE), and trimethoxy(1H,1H,2H,2H-heptadecafluorodecyl)silane (TMHFS) using a layer-by-layer method. The results indicated that the LWAP exhibited a breakage ratio of 2% under 52 MPa (7.5 K) closure stress, with an apparent density of 2.12 g/cm^3^ and a bulk density of 1.21 g/cm^3^. The contact angles of water and olive oil were 125° and 104°, respectively, changing to 124° and 96° after displacement by water and diesel oil. A comparison showed that the LWAP could transport over a significantly longer distance than the LWCP, with the length increasing by more than 80%. Meanwhile, the LWAP displayed notable resistance to scale deposition on the proppant surface compared to the LWCP. Furthermore, the maintained conductivity of the LWAP was higher than that of the LWCP after displacement by water and oil phases alternately. The modified proppant could minimize production declines during hydrocarbon extraction in unconventional reservoirs.

## 1. Introduction

Due to the significant increase in energy demands, the exploitation of unconventional oil and gas resources has become a central focus of energy development strategies in numerous countries [1,2,3]. Horizontal drilling techniques, combined with multistage hydraulic fracturing, have facilitated the development of substantial oil and gas reserves from unconventional formations [4]. In hydraulic fracturing operations, the proppant plays an essential role in keeping the fractures created in the rock formation open. By injecting a proppant along with the fracturing fluid, highly permeable fractures are created, enabling the efficient flow of oil and gas to the wellbore [5,6].

The proppant serves as the fundamental material and is essential in enhancing the stimulation effect [7,8]. The characteristics of the proppant utilized in hydraulic fracturing significantly influence the well productivity [9]. Presently, the proppants employed in fracturing operations include quartz sand, ceramic proppants, and coated proppants [10]. To enhance the construction safety, reliability, and production within the oilfield sector, hydraulic fracturing technology has garnered significant attention from both academia and industry [11]. As a result, many studies have been undertaken to enhance the performance of the proppant using techniques like surface modification and density reduction [12,13,14,15,16,17].

A superhydrophobic proppant has been evaluated, which demonstrates that superhydrophobic surfaces reduce gel damage from fracturing fluids, enhance the conductivity, and decrease the well cleanup times [18]. However, it has not been proven that a superhydrophobic modified proppant can enhance the fluid flow of both water and oil in the proppant pack, which could lead to an optimized dual-phase flow [19,20].

If water and oil do not adhere to the surface of a neutrally wet proppant, this can not only decrease water saturation but also facilitate oil movement, as highlighted by Bestaoui-Spurr [21]. Consequently, the pores remain fully open for the phases to flow through, and the capillary pressure is virtually zero for both phases. Moreover, a neutral wet surface reduces the intermolecular forces between the surfaces and the liquid phases, facilitating the flow of these fluids to the wellbore.

In recent years, Bestaoui-Spurr and colleagues introduced a neutral wet proppant and applied it in several fracture stimulations [21,22,23,24]. This new proppant enabled a significantly high flowback rate, allowing hydrocarbons to start flowing sooner than expected and at a lower drawdown pressure. Furthermore, wells with a very low bottomhole pressure that were stimulated with a neutral wet proppant cleaned up much faster compared to those using a conventional proppant [20]. Currently, research on neutral wet proppants mainly focuses on their effects on the fracturing fluid backflow and well production, while there is a significant lack of information on fabrication techniques and thorough testing.

Amphiphobic surfaces, which feature high surface roughness and low surface energy, effectively repel liquids with low surface tension. By creating micro-/nanostructures on membrane surfaces and introducing low surface energy, it is possible to transition from the fully wetted Wenzel state to the Cassie–Baxter state [25]. Among various hydrophobic materials, polytetrafluoroethylene (PTFE) is notable for its excellent hydrophobic properties, high chemical inertness, and other advantageous characteristics [26]. Low-surface-energy PTFE particles are utilized to create rough structures, and surface fluorination with fluoroalkyl molecules further reduces the surface energy. Phenolic resins and epoxy resins provide outstanding mechanical performance, excellent solvent and corrosion resistance, high heat resistance, superior water resistance, cost-effectiveness, and remarkable compatibility with various substrates. They are widely used in coatings [27,28].

In this study, we report a straightforward method for the manufacturing of LWAPs using common materials. We also aimed to thoroughly investigate the benefits of the LWAP and its hydrophobic and oleophobic mechanisms through experimentation. Additionally, some innovative testing methods, such as measurements of the amphiphobic durability, maintained conductivity, and scale resistance, were employed to understand the unique properties of the LWAP, which demonstrated outstanding performance.

## 2. Materials and Methods

### 2.1. Materials

PTFE with a particle size of 3 μm was purchased from DuPont Co. (Shenzhen, China). Trimethoxy(1H,1H,2H,2H-heptadecafluorodecyl)silane (TMHFS) was purchased from Rhawn Chemical Technology Co. (Shanghai, China). Calcium chloride, sodium dodecyl sulfate, and sodium bicarbonate were purchased from Titan Scientific Co. (Shanghai, China). LWCP 20/40 mesh, phenol resin (PF1901), epoxy resin (E51), hexamethylenetetramine (Hexa), and a modified phenolic amine curing agent (MPACA) were provided by Bei Jing Kun Lun Long Yuan Petroleum Exploitation Technology Co. (Beijing, China). 

### 2.2. Experimental Procedure

The proppant coating process was conducted in two stages. Initially, PF1901 was blended into a pre-heated LWCP (T = 170 °C, LWCP–PF1901 = 100:1) and stirred for 30 s. Subsequently, a solution of Hexa with a 40% concentration (PF1901: 40% Hexa = 2:1) was added and the mixture was stirred for another 30 s. Then, epoxy resin (LWCP–E51 = 100:1) was mixed in and stirred for 30 s, followed by the introduction of an MPACA and PTFE (E51: MPACA–PTFE = 10:2:20). These were incorporated and stirred for 1 min. The mixture was then heated in an oven at 100 °C for 2 h to create the initial resin layer.

**Figure 1:**
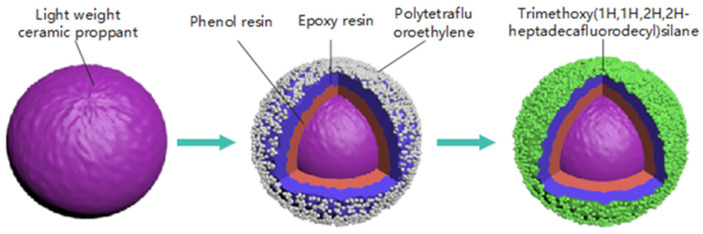
Schematics of the modification process.

The second coating stage involved coating the primed proppant with TMHFS. TMHFS (PTFE–TMHFS = 2:1) was coated onto the primed proppant surface using a simple stirring method at room temperature. The sample was then heated in an oven at 55 °C for 6 h to ensure the formation of an amphiphobic layer on the surface of the primed proppant. The proppant was screened to remove coarse and fine particles. This process is illustrated in Figure 1, and the raw material composition of the LWAP is shown in Table 1.

### 2.3. Test Methods

#### 2.3.1. Basic Performance

The basic performance of the proppant was analyzed according to the Chinese Petroleum and Gas Industry Standard “Measurement of properties of proppants used in hydraulic fracturing and gravel-packing operations” (SY/T 5108-2014) [29].

The turbidity test procedure aims to determine the amount of suspended particles or other finely divided matter present. The steps are as follows. Measure 40.0 g of a dry proppant sample and add it, along with 100 mL of demineralized water, to a 250 mL wide-mouth bottle. Allow the mixture to stand for 30 min. Then, shake it horizontally back and forth by hand for 0.5 min, performing 60 shakes, and allow it to stand for an additional 5 min. Using a syringe, extract water from near the center of the water volume, taking care not to extract any proppant particles, as this could distort the results. Place the water sample in a test vial and insert it into a calibrated turbidity meter. Record the sample’s turbidity in NTU.

#### 2.3.2. Conductivity

The conductivity of the proppant is defined as the product of the width of the proppant pack and its permeability, as described in the process outlined in “The Procedures for Measuring the Conductivity of Proppants” (SY/T 6302-2019) [30]. The calculation formula at Darcy flow conditions is shown in Equation (1).

(1) $$kW_{f}=\frac{99.998\mu QL}{\omega \Delta p}$$

where 

*kW_f_* is the conductivity of the proppant pack, μm^2^·cm; 

*k* is the proppant pack’s permeability, μm^2^;

*W_f_* is the pack width;

*µ* is the viscosity of the test liquid at the test temperature, cP; 

*Q* is the flow rate, cm^3^/min;

*L* is the length between the pressure ports, cm; 

ω is the cell width, cm^2^; 

Δ*p* is the pressure difference between upstream and downstream, kPa.

#### 2.3.3. Wettability

The wettability of the LWAP was evaluated by measuring the static contact angle on its surface using a contact angle meter (Dataphysics OCA50) at room temperature. Prior to the test, LWAP samples of 20/40 mesh size (850–425 μm) were firmly placed on a double-sided adhesive to ensure a dense single-layer structure [31]. Then, we added 5 μL of test liquid dropwise onto the surface, measured it, and took photos. We conducted three tests at different points and took the maximum value.

The amphiphobic durability of the LWAP was assessed using the following method. Initially, 20 mL of a dry proppant sample was measured and placed in a square plexiglass tube (10 × 20 × 500 mm), which was sealed at the bottom with a sieve. The proppant was then saturated with water and soaked for 24 h, followed by the addition of 40 mL diesel oil into the tube upon fluid exhaustion, with this process repeated twice. Subsequently, the proppant was removed and dried in an oven at 60 °C for 2 h. The contact angle on its surface was then evaluated. 

#### 2.3.4. Transport Performance

**Figure 2:**
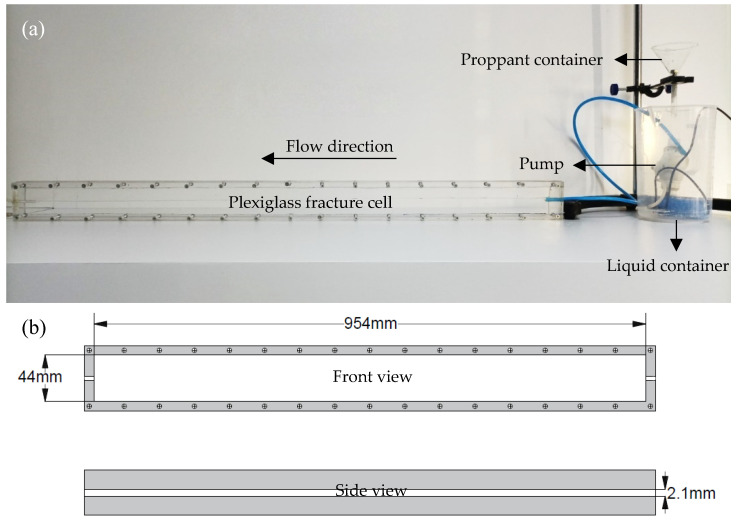
(**a**) Photograph of fracture model apparatus; (**b**) schematic of plexiglass fracture cell (not to scale).

As part of the testing, a fracture model test was conducted to compare the transport performance of the proppant. Figure 2a shows the fracture model apparatus used to measure the settled proppant. The fracture cell was composed of plexiglass with two smooth, parallel walls. The cell measured 954 mm in length and 44 mm in width, with wall spacing of 2.1 mm, as shown in Figure 2b.

The procedure is as follows. Initially, add a sodium dodecyl sulfate solution with a concentration of 2 ppm to the liquid container, ensuring that the liquid level exceeds the upper inlet of the pump. Subsequently, activate the pump to fill the model with the liquid. Next, introduce 40 mL of the test proppant into the proppant container, open the discharge port, initiate timing, and cease both the pump operation and timing when the cell inlet contains pure liquid. Throughout the experiment, continuously replenish the sodium dodecyl sulfate solution to ensure that the liquid level remains above the pump port. The clear water flow rate of the pump was 760 mL/min.

#### 2.3.5. Scale Resistance 

In this research, calcium chloride and sodium bicarbonate solutions were utilized to assess the efficacy of the LWAP in combating the calcium carbonate scale. Initially, 5.0 g of a dried proppant sample was measured and transferred to a 250 mL glass beaker with 10 mL of 0.5 mol/L calcium chloride solution, and they were mixed thoroughly. Subsequently, 10 mL of 0.5 mol/L sodium bicarbonate solution was added and they were mixed well. The sample and fluid were then allowed to soak in the beaker for 4 days at room temperature before being transferred to a 70 sieve. The sample was rinsed with water and dried at 60 °C until a constant weight was achieved. Finally, the surface morphologies and compositions of the proppant were analyzed using scanning electron microscopy (SEM) and an energy-dispersive spectrometer (EDX) attached to the SEM.

#### 2.3.6. Maintained Conductivity

Conductivity measurements were conducted post-displacement to evaluate the maintained conductivity of the proppant pack following the flow of water and oil phases. Initially, conductivity data were obtained using 5 kg/m^2^ samples under 40 MPa closure stress and at room temperature to establish the baseline conductivity. Subsequently, the maintained conductivity was assessed after displacing the water and oil phases, with DI water utilized as the flowing fluid. It is essential to maintain consistent timing for the comparative experiment before measuring the conductivity.

## 3. Results and Discussion

### 3.1. Physical and Chemical Properties

To investigate the effects of the coating on the LWCP, standard proppant tests were performed. The particle size distribution and basic performance parameters of proppants that meet the requisite standards are presented in Table 2 and Table 3. 

As expected, the findings verified that the coating improved the basic characteristics of the proppant, notably reducing the density, breakage ratio, and turbidity significantly. The breakage ratio was 2% under closure stress of 52 MPa (7.5 K), with an apparent density of 2.12 g/cm^3^ and a bulk density of 1.21 g/cm^3^. The low-density feature of the LWAP ensures that the proppant travels further in fractures, thus maintaining the conductivity of hydraulic fractures [32,33]. Additionally, the low turbidity and breakage ratio contribute to the maintained conductivity of the proppant [34].

**Figure 3:**
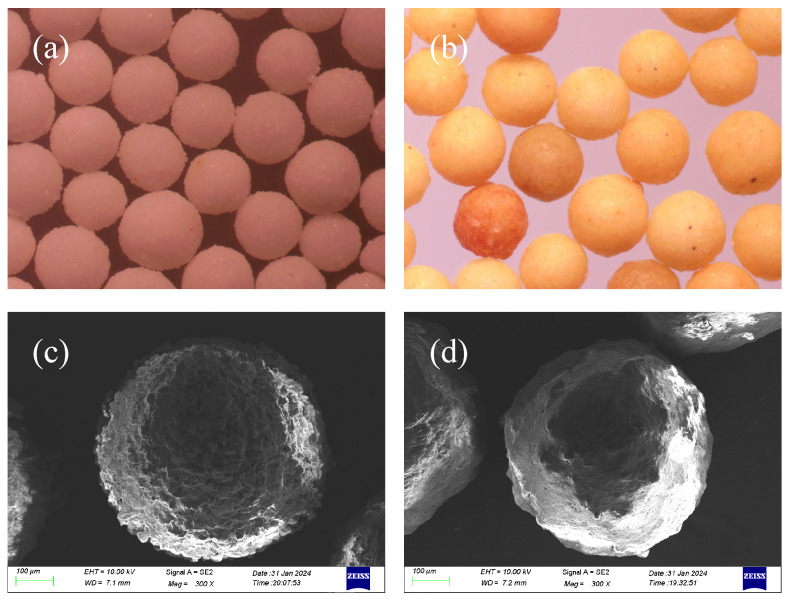
Optical and SEM images of proppants: (**a**,**c**) LWCP; (**b**,**d**) LWAP.

Optical images of the LWCP and LWAP were captured with a digital camera and are displayed in Figure 3a,b. The color of the LWAP grains appeared distinct from those of the untreated LWCP, with the difference attributed to the coating chemicals.

Moreover, SEM images of the LWCP and LWAP were captured with a ZEISS GeminiSEM 300 and are displayed in Figure 3c,d. The surfaces of the LWAP grains appear distinct from those of the untreated LWCP. The LWCP exhibits a dense convex structure. In contrast, while a limited number of protrusions can be observed on the surface of the LWAP, it remains relatively smooth. This smoothness is attributed to the coating layer filling the low-lying areas between the protrusions.

### 3.2. Wettability and Durability

#### 3.2.1. Wettability 

**Figure 4:**
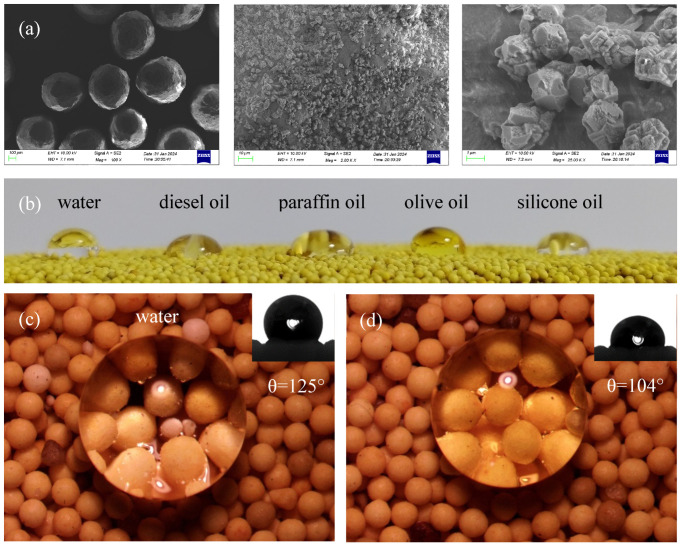
(**a**) SEM images of various scales of LWAP grains; (**b**) images of liquid drops on LWAP; (**c**) images of water drop on LWAP; (**d**) images of olive oil drop on LWAP.

The SEM images, presented in Figure 4a, show the LWAP grains produced through a simple stirring method, exhibiting a rough surface composed of micro- and nanoparticles. Notably, the micro-PTFE particles are firmly attached to the surface of the proppant grains, featuring nanostructures. 

To assess the effectiveness of the surface modification, droplets of five different liquids were placed on the proppant’s surface. The liquid droplets on the proppant surface showcase the amphiphobic nature of the 20/40 mesh LWAP, as shown in Figure 4b. Additionally, the contact angle was measured using the sessile drop technique. As demonstrated in Figure 4c,d, the LWAP exhibits outstanding amphiphobic properties, repelling both water and oil, with contact angles of 125° and 104° for water and olive oil, respectively. This behavior is attributed to the surface’s roughness and the presence of materials with low surface energy [35].

The amphiphobic nature of the LWAP helps to decrease the capillary forces, preventing the entrapment of oil or water in the proppant pack. This ensures that the pore throats remain unobstructed and enhances the overall conductivity.

**Figure 5:**
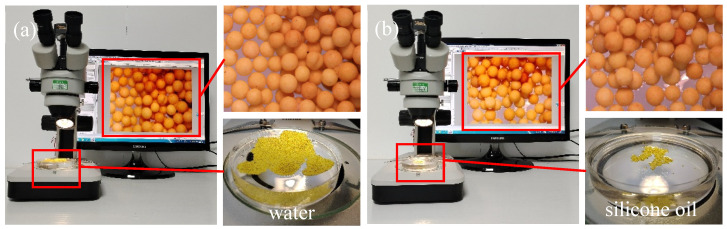
Images of LWAP suspended on the surfaces of different liquids; (**a**) LWAP suspended on the surfaces of water; (**b**) LWAP suspended on the surfaces of silicone oil.

In order to evaluate the suspension performance of the LWAP, it was poured into the fluid to observe the phenomenon. It can be seen from Figure 5a,b that the suspension effect of the LWAP in water and silicone oil is excellent. This is attributed to the low density and amphiphobic nature of the LWAP. As stated by Chen [36], the distance of proppant migration in unconventional reservoir fractures can be directly reflected by the suspension effect of the proppant in the fluid. Proppants with a high capacity for suspension can be transported by the fluid to the fracture tip, increasing the length of the propping fracture and having a better proppant distribution in the fracture.

#### 3.2.2. Amphiphobic Durability

**Figure 6:**
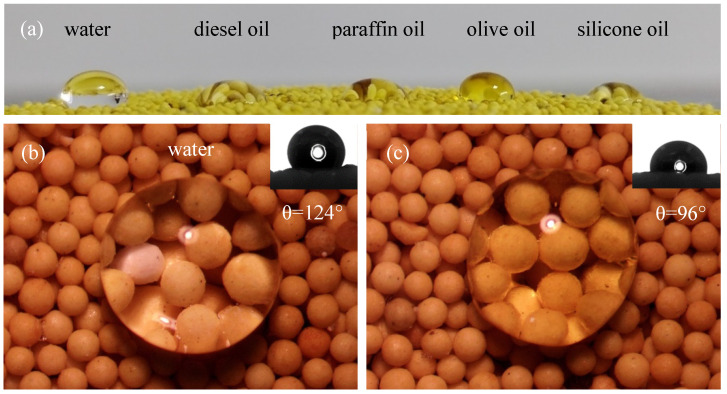
Liquid drops on LWAP under displacement with water and oil; (**a**) images of liquid drops on LWAP; (**b**) images of water drop on LWAP; (**c**) images of olive oil drop on LWAP.

We conducted alternating displacement experiments to determine the durability of the modified LWAP’s surface. After displacement with water and diesel oil alternately, the LWAP grains still maintained good amphiphobicity (Figure 6a). Additionally, the contact angles for water and olive oil were measured at 124° and 96°, respectively, as shown in Figure 6b,c. 

These properties effectively ensure that the proppant remains amphiphobic even after entering the fracture, reducing the risk of oil and water blocking the pore throat and ensuring the conductivity of the proppant pack.

### 3.3. Transport Performance

**Figure 7:**
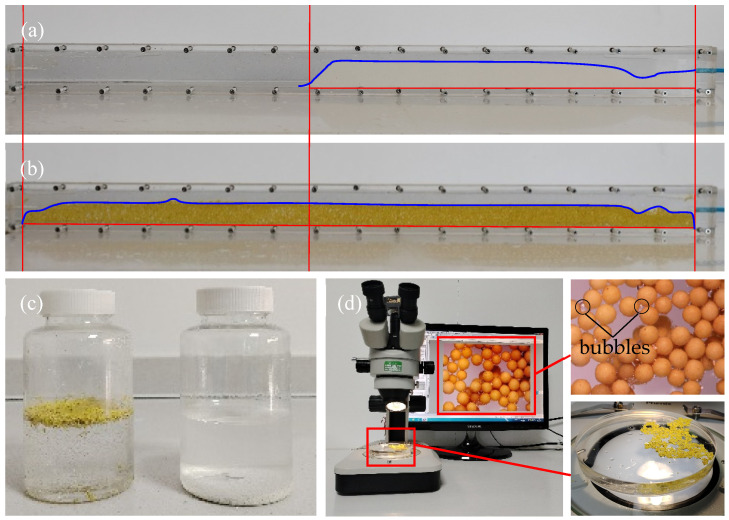
Images of proppant placement in fracture systems and the state in water after stirring (2 ppm sodium dodecyl sulfate solution): (**a**) LWCP placement in fracture system; (**b**) LWAP placement in fracture system; (**c**,**d**) the state in water after stirring. (The blue lines in (**a**,**b**) are auxiliary curves representing the settled proppant bed heights; The red lines in (**a**,**b**) are auxiliary curves representing the settled proppant bed heights; The area between the right and middle red lines (**a**,**b**)represents the length of the LWCP settling bed, and the area between the right and left red lines (**a**,**b**) represents the length of the LWAP settling bed.).

As part of the performance testing, a fracture cell test was conducted to compare the transport efficiency of the proppants using a 2 ppm sodium dodecyl sulfate solution. The plexiglass fracture cell schematic was utilized to measure the proppant’s settled bed height and bed length. These parameters were employed to assess and contrast the transport efficiency for the LWCP and LWAP 20/40 proppants. Figure 7a,b depict the testing results, confirming the enhanced transport capabilities of the treated LWAP in comparison to the untreated LWCP.

Several observations were noted during the testing. While flowing, a significant amount of the LWAP was seen floating. However, a bank eventually formed. It was observed that this bank was notably lower and more voluminous compared to the LWCP. The LWAP pack height decreased by over 18%, and the length of the LWAP pack increased by over 80%. Furthermore, the resulting dune appeared more porous than the harder packed dune seen with the LWCP. Based on the tested data and bulk density, it can be calculated that the porosity of the LWAP pack increased by more than 50%. These changes can be attributed to the unique properties of the LWAP. When the LWAP is added to the fluid, gas is simultaneously introduced between the pores of the proppant particles. Due to the hydrophobicity of the LWAP particles, the contact between their surfaces and the liquid is in the Cassie–Baxter state, causing bubbles to adhere to the surface of the proppant. During the pumping process, with the stabilizing effect of sodium dodecyl sulfate, gas is pumped in along with the proppant and liquid without being compromised, ultimately forming porous filling that improves the distribution of the proppant within the cell.

To investigate the proppant suspension properties at high shear rates to simulate the environment during stimulation, a blender test was conducted. The proppant was added to a 2 ppm sodium dodecyl sulfate solution and exposed to high shear rates. As depicted in Figure 7c (left LWAP, right LWCP), the LWAP was able to remain suspended in the 2 ppm sodium dodecyl sulfate solution. In contrast, the LWCP settled at the bottom of the container. To comprehend the cause, we further examined the suspended LWAP under a microscope, which was agitated at high shear rates. As illustrated in Figure 7d, it is evident that bubbles adhered to the surfaces of the LWAP particles. This results in a reduction in particle density, combined with the surface’s roughness and the presence of materials with low surface energy, thereby facilitating suspension.

Interestingly, we can find in Figure 7b that the proppant settled and formed a sand dam within the cell, rather than remaining suspended as shown in Figure 7d. During the pumping experiments, the LWAP only entrained the gas present between the particles into the system, resulting in a relatively small amount of gas being introduced during pumping. This differs from the stirring process, where particles can repeatedly come into contact with the air and adhere to air bubbles. Additionally, the width of the cell is only 2.1 mm, while the mean diameter of the proppant is 0.735 mm. Consequently, during the pumping process, only two to three proppant particles can pass through simultaneously, disrupting the suspended units composed of the proppant and gas. This disruption causes some bubbles to detach from the surface of the proppant or coalesce into larger bubbles, leading to a significant reduction in the amount of gas adhered to the particles, which inhibits the formation of a suspended system. The combined effect of these two factors results in the proppant settling and forming a porous sand dam within the cell, rather than remaining suspended. This characteristic prevents the proppant from being returned with the flowback fluid during the backflow process, which would otherwise lead to insufficient support for fractures due to the proppant’s inability to settle and form a sand dam.

These characteristics allow for the deeper penetration of the LWAP into the intricate fracture network and promote better proppant distribution within the fracture. Consequently, by strategically situating the proppant effectively and enhancing its volume within the fracture, production is enhanced by improving the conductive fracture network.

### 3.4. Anti-Scaling Property

**Figure 8:**
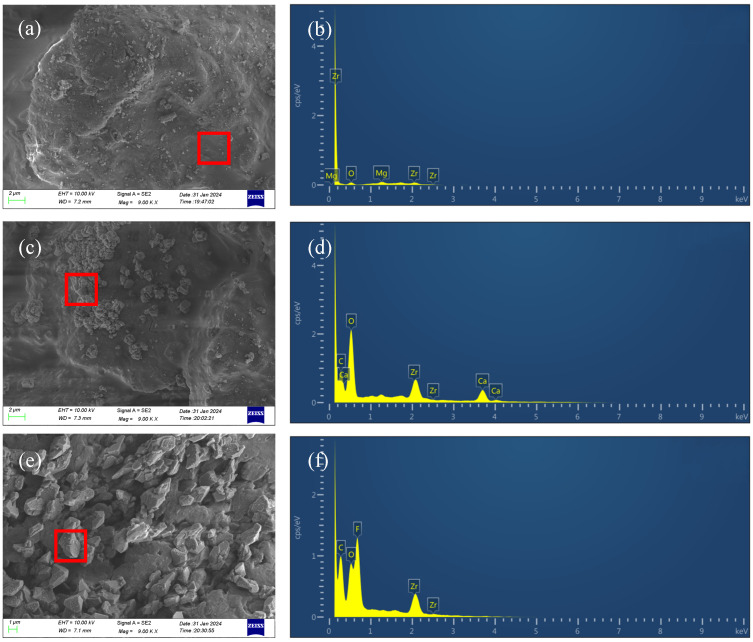
SEM images and EDX of proppant: LWCP (**a**,**b**); LWCP immersed in brine (**c**,**d**); LWAP immersed in brine (**e**,**f**). (The red boxes in (**a**,**c**,**e**) are the EDX spectral scanning sampling areas).

By comparing the SEM images before and after soaking, it is evident that no large crystal areas were present on the LWCP surface before soaking (Figure 8a). However, cubic crystals emerged on the LWCP surface after soaking (Figure 8c). Comparing the EDX spectra of the surface crystals of the LWAP particles after soaking (Figure 8d) with the surfaces of the ceramic particles before soaking (Figure 8b), it is noticeable that calcium and carbon appeared post-soaking. Hence, it can be deduced that calcium carbonate crystals were deposited on the ceramic support agent’s surface. 

Comparing the scanning electron microscopy of the LWAP before (Figure 4a) and after soaking (Figure 8e), it can be observed that there is no significant change on the particle surface. Additionally, the EDX analysis reveals that the irregular particles on the support agent’s surface consist mainly of fluorine, carbon, and oxygen (Figure 8f). Therefore, it can be inferred that the particles adhering to the LWAP are PTFE particles. Since no calcium was detected in the analysis, it can be concluded that the LWAP effectively prevents calcium carbonate deposition on its surface, ensuring pore throat smoothness and conductivity.

### 3.5. Conductivity

**Figure 9:**
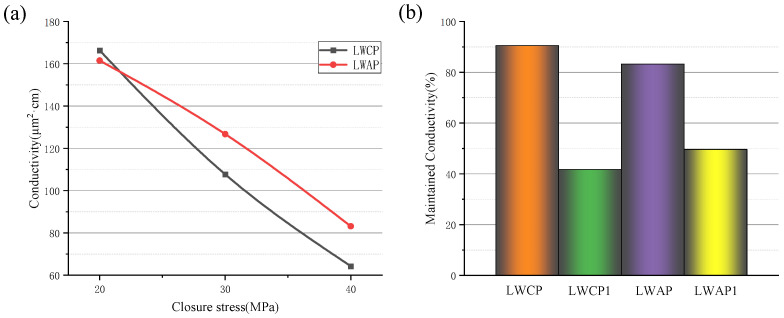
Conductivity (**a**) and maintained conductivity under 40 MPa (**b**) of different proppants using DI water as a flowing fluid (LWCP1 and LWAP1 after water and oil displacement).

The conductivity data in Figure 9a show water as the flowing fluid. As expected, the conductivity decreases with increasing stress. A comparison is performed between the conductivity of the 20/40 LWAP and that of the control LWCP 20/40. The conductivity of the LWAP is initially lower than that of the control, but, when the pressure reaches about 22 MPa, the two curves intersect, and the conductivity of the LWAP becomes greater than that of the LWCP. The improved conductivity is primarily attributed to the increased strength of the LWAP and the reduction in fine transport in the proppant pack.

Conductivity measurements were taken before and after displacement to determine the maintained conductivity of the proppant pack following fracturing fluid cleanup and hydrocarbon flow. The proppants used for comparison were the 20/40 LWAP and 20/40 LWCP. Initially, the conductivity of both proppants was measured with water for 25 min, and the average of the last three numbers was taken to establish the baseline conductivity. Subsequently, displacement was carried out with water for approximately 60 min, and the average of the last three numbers represented the conductivity after water displacement. The maintained conductivity ratios of the LWCP and LWAP were found to be 90.5% and 83.2%, as depicted in Figure 9b.

Furthermore, the conductivity of both proppants was measured with water for 25 min to establish the baseline conductivity. Subsequently, displacement was conducted with diesel oil for 30 min, followed by an immediate switch to water displacement for around 30 min. After stabilization, the last three numbers were taken to determine the conductivity of the proppant after oil and water displacement. The rate of maintained conductivity was calculated by dividing the displacement conductivity by the baseline conductivity. The maintained conductivity ratios after the alternating displacement of the LWCP and LWAP were found to be 41.7% and 49.6%, respectively (Figure 8b).

The benefits of the LWAP are due to its amphiphobic nature, reducing the likelihood of attracting oil and water. This will not create a film of water or oil that keeps the pore throats clear, potentially improving the conductivity.

## 4. Conclusions

In summary, a lightweight amphiphobic proppant was successfully manufactured. The proppant exhibits excellent amphiphobicity both before and after exposure to various fluids, attributed to its rough surface composed of low-surface-energy material. Furthermore, the LWAP can penetrate deeper into the fracture network, has better proppant distribution in the fracture, and demonstrates notable improvements in its conductivity performance after the flow of water and oil phases compared to the LWCP.

## Figures and Tables

**Table 1:** Raw material composition of LWAP.

Raw Materials	LWCP	PF1901	Hexa	E51	MPACA	PTFE	TMHFS
Percentage, wt%	94.88	0.95	0.19	0.95	0.19	1.9	0.95

**Table 2:** Particle size distribution.

Sieve Size	1180 μm	850 μm	710 μm	600 μm	500 μm	425 μm	300 μm	Pan
LWCP	0.00	1.51	59.99	38.25	0.25	0.00	0.00	0.00
LWAP	0.00	2.37	76.18	20.96	0.49	0.00	0.00	0.00

**Table 3:** Basic performance parameters of proppants.

Parameter	LWCP	LWAP
Bulk density, g/cm^3^	1.34	1.21
Apparent density, g/cm^3^	2.48	2.12
Roundness	0.9	0.9
Sphericity	0.9	0.9
Turbidity, NTU	88	12
Acid solubility, %	5.6	3.2
Breakage ratio under 52 MPa (7.5 K), %	8	2

## Data Availability

The original contributions presented in the study are included in the article, further inquiries can be directed to the corresponding authors.
